# Rule-based and machine learning algorithms identify patients with systemic sclerosis accurately in the electronic health record

**DOI:** 10.1186/s13075-019-2092-7

**Published:** 2019-12-30

**Authors:** Lia Jamian, Lee Wheless, Leslie J. Crofford, April Barnado

**Affiliations:** 1 Hartford HealthCare Medical Group, Hartford, CT USA; 2 Department of Dermatology, Vanderbilt University Medical Center, Nashville, TN USA; 3 Data Science Institute, Vanderbilt University Medical Center, Nashville, TN USA; 4 Department of Medicine, Vanderbilt University Medical Center, 1161 21st Avenue South T3113 MCN, Nashville, TN 37232 USA

**Keywords:** Systemic sclerosis, Electronic health records, Algorithms, Bioinformatics

## Abstract

**Background:**

Systemic sclerosis (SSc) is a rare disease with studies limited by small sample sizes. Electronic health records (EHRs) represent a powerful tool to study patients with rare diseases such as SSc, but validated methods are needed. We developed and validated EHR-based algorithms that incorporate billing codes and clinical data to identify SSc patients in the EHR.

**Methods:**

We used a de-identified EHR with over 3 million subjects and identified 1899 potential SSc subjects with at least 1 count of the SSc ICD-9 (710.1) or ICD-10-CM (M34*) codes. We randomly selected 200 as a training set for chart review. A subject was a case if diagnosed with SSc by a rheumatologist, dermatologist, or pulmonologist. We selected the following algorithm components based on clinical knowledge and available data: SSc ICD-9 and ICD-10-CM codes, positive antinuclear antibody (ANA) (titer ≥ 1:80), and a keyword of Raynaud’s phenomenon (RP). We performed both rule-based and machine learning techniques for algorithm development. Positive predictive values (PPVs), sensitivities, and *F*-scores (which account for PPVs and sensitivities) were calculated for the algorithms.

**Results:**

PPVs were low for algorithms using only 1 count of the SSc ICD-9 code. As code counts increased, the PPVs increased. PPVs were higher for algorithms using ICD-10-CM codes versus the ICD-9 code. Adding a positive ANA and RP keyword increased the PPVs of algorithms only using ICD billing codes. Algorithms using ≥ 3 or ≥ 4 counts of the SSc ICD-9 or ICD-10-CM codes and ANA positivity had the highest PPV at 100% but a low sensitivity at 50%. The algorithm with the highest *F*-score of 91% was ≥ 4 counts of the ICD-9 or ICD-10-CM codes with an internally validated PPV of 90%. A machine learning method using random forests yielded an algorithm with a PPV of 84%, sensitivity of 92%, and *F*-score of 88%. The most important feature was RP keyword.

**Conclusions:**

Algorithms using only ICD-9 codes did not perform well to identify SSc patients. The highest performing algorithms incorporated clinical data with billing codes. EHR-based algorithms can identify SSc patients across a healthcare system, enabling researchers to examine important outcomes.

## Introduction

Systemic sclerosis (SSc) is a rare, chronic, autoimmune disease with high morbidity and mortality. Due to its rarity, SSc studies are limited by sample size, with most being single center cohort studies, making it difficult to assess outcomes and comorbidities. To study any disease, there must be an efficient and accurate way to identify individuals with the disease. While prospective cohort studies longitudinally follow a cohort, they can be expensive and time consuming. Administrative claims databases can serve as an efficient and cost-effective way to study a large, diverse cohort. Data from these databases, however, are limited by the accuracy of billing codes and a lack of granular clinical data. The electronic health record (EHR) can bridge these limitations by including longitudinal and detailed data on medications, labs, and comorbidities [1].

The widespread implementation of electronic health records (EHRs) has enabled the integration of large amounts of patient data across diverse healthcare settings and populations [1]. The EHR can function as a practical tool to study rare diseases longitudinally such as SSc. Methods to accurately identify patients with SSc in the EHR have not been fully developed. Two studies aimed to identify SSc patients in the EHR. One specifically focused on identifying patients at risk for SSc renal crisis in a veteran’s population [2], and the other only examined the performance of a one-time International Classification of Disease Ninth Version (ICD-9) billing code to identify patients with SSc [3]. While ICD billing codes are often used to define disease cohorts, this method may not accurately identify patients with autoimmune diseases [4, 5]. We sought to use clinically meaningful variables readily available in the EHR that would broadly capture SSc patients across the healthcare system. Specifically, we developed and validated algorithms that incorporate ICD-9 and ICD-10-CM codes, laboratory data, and keywords to accurately identify SSc patients in the EHR.

## Methods

### Subject selection

Following approval from the Vanderbilt University Medical Center (VUMC) Institutional Review Board, we analyzed data from a de-identified version of Vanderbilt’s EHR called the Synthetic Derivative [1]. The Synthetic Derivative contains all available clinical data from Vanderbilt’s EHR with over 3 million subjects with longitudinal follow-up over several decades. The Synthetic Derivative contains all Vanderbilt inpatient and outpatient notes, laboratory values, diagnostic and procedure billing codes, demographics, imaging, and pathology reports. Scanned documents from other healthcare systems are not available in the Synthetic Derivative. The Synthetic Derivative interface allows one to efficiently search records using demographics, ICD codes, and keywords.

**Fig. 1:**
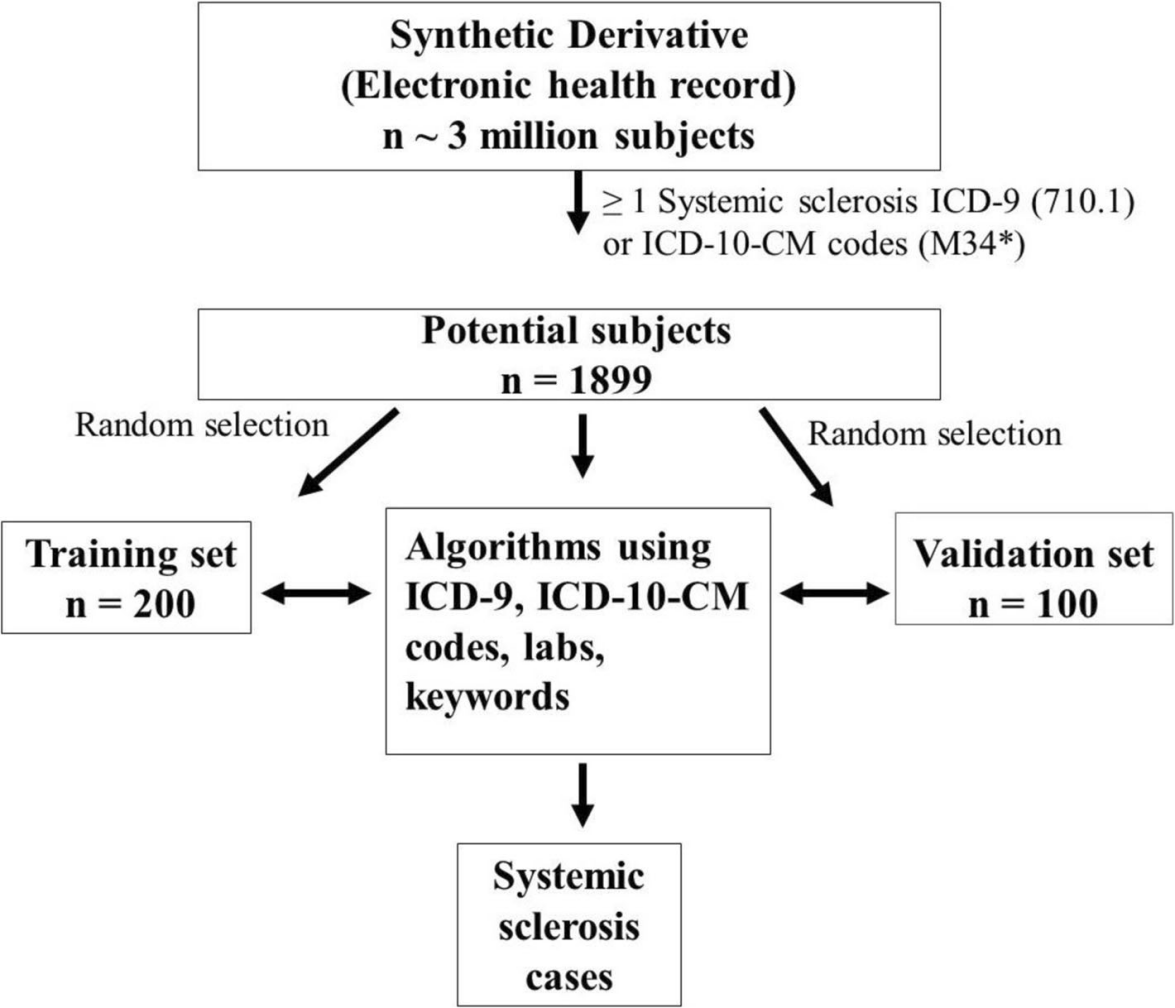
Development of algorithms to identify patients with systemic sclerosis (SSc) in the electronic health record (EHR). At least a 1-time count of the SSc ICD-9 code (710.1) or ICD-10-CM codes (M34*) was applied to the 3 million subjects in Vanderbilt’s Synthetic Derivative, which resulted in 1899 potential SSc cases. Of these 1899 potential SSc cases, 200 were randomly selected for a training set to develop and test algorithms with various combinations of the SSc ICD-9 and ICD-10-CM codes, keyword search for Raynaud’s phenomenon, and positive ANA (≥ 1:80). The highest performing algorithm was internally validated in a set of 100 subjects who were not part of the original training set

Using the Synthetic Derivative, we identified potential SSc cases with at least 1 count of the SSc ICD-9 (710.1) or ICD-10-CM (M34*) codes, totaling 1899 subjects (Fig. 1). We then randomly selected 200 of these subjects as a training set for chart review to identify their true case status. Chart review of the 200 potential SSc patients was conducted by a rheumatologist (LJ). A random 50 of the 200 potential cases were reviewed by another rheumatologist (AB), who was blinded to the initial rheumatologists’ verdict of case status to determine interrater reliability. A subject was defined as a case if diagnosed with SSc by a Vanderbilt or external rheumatologist, dermatologist, or pulmonologist (specialists). Subjects with sine scleroderma, overlap syndrome with SSc features (i.e., SSc with rheumatoid arthritis or Sjogren’s syndrome), or mixed connective tissue disease (MCTD) with SSc features were included as cases. Subjects with morphea were not included as cases. If diagnosed by a specialist outside of Vanderbilt, the external specialist was required to be clearly mentioned in available notes. During chart review, potential subjects were classified as (1) cases, (2) not cases with alternative diagnoses noted, (3) unconfirmed cases if there was uncertainty in the diagnosis, or (4) missing if there was missing clinical documentation. In the analysis, subjects with uncertainty in the SSc diagnosis were counted as not cases. Missing subjects were excluded from analyses. Race/ethnicity data from the Synthetic Derivative is a combination of self-report and administrative entry. These data have previously been validated and highly correlate with genetic ancestry [6].

### Algorithm development

A priori, we selected the following algorithm components using an expert panel of rheumatologists based on clinical knowledge and accessible data in the EHR: ICD-9 (710.1) and ICD-10-CM (M34*) codes for SSc, positive antinuclear antibody (ANA) (titer ≥ 1:80), and a keyword of Raynaud’s phenomenon (RP) in clinical notes. PPVs and sensitivities were calculated for combinations of the above algorithm components using “and” or “or” to connect algorithm components. If ANA testing was not performed at VUMC, it was considered missing, and that subject’s data were not used. For the RP keyword, a keyword search was conducted across all inpatient and outpatient notes using natural language processing. Chart review was then performed to ensure the keyword captured the presence and not the absence of RP.

The occurrences of billing codes represent distinct days. For example, if a subject has multiple billing codes for SSc on 1 day (i.e., clinic visit, lab encounter, radiology), only one SSc code is recorded for that day. For algorithms using the ICD-10-CM codes, a subject had to have at least one ICD-10-CM code for any condition to be included in the analysis. Positive predictive value (PPV), sensitivity, and an *F*-score were calculated for each of the algorithms. PPV was defined as the number of subjects who fit the algorithm that were confirmed cases on chart review divided by the total number of subjects who fit the algorithm. Sensitivity was defined as the number of subjects who fit the algorithm that were confirmed cases on chart review divided by the total number of confirmed cases. The subjects who were included in the PPV and sensitivity analyses were required to have complete data for all components of the algorithm being tested. The *F*-score, which is the harmonic mean of the PPV and sensitivity ([2 × PPV × sensitivity]/[PPV + sensitivity]), was calculated for all algorithms. The *F*-score is commonly used in bioinformatics to compare algorithms, as it accounts for both PPV and sensitivity.

### Classification and regression tree and random forest modeling

In addition to traditional, rule-based algorithms, described above, we employed machine learning methods to generate data-driven EHR algorithms. For these methods, the user controls input variables but the learning process for the algorithm development is somewhat of a “black box.” Specifically, we used classification and regression tree (CART) and random forest (RF) for algorithm development [7, 8]. CART is a modeling technique that builds a classification tree using all the input variables simultaneously. RF is an extension of CART that builds classification trees using a random sample of the input variables. This random sample is then done multiple times, thus creating a “forest” of classification trees. The final classification is the average of the forest. The caret package in R was used for model tuning to identify the best model parameters, including number of variables per sample, minimum node size, and splitting rules. Within caret, the rpart and ranger packages were used to conduct CART and random forest analyses, respectively. All analyses were conducted in R version 3.5.1.

### Algorithm validation

We validated the algorithm with the highest *F*-score in a set of 100 randomly selected subjects. These random subjects were from the 1899 subjects with at least 1 count of the ICD-9 or ICD-10-CM SSc codes but were not in the training set (Fig. 1).

### Statistical analyses

We assessed for differences between subjects who met the case definition for SSc versus those who did not using the Mann-Whitney *U* for continuous variables, as there were non-normal distributions in the data, and chi-square or Fisher’s exact test for categorical variables. Our null hypothesis was that there would be no difference in the PPVs of algorithms that incorporate lab values and RP keyword with SSc ICD-9 or ICD-10-CM codes compared to algorithms that use only SSc ICD-9 or ICD-10-CM codes. Our preliminary data showed that using two counts of the SSc ICD-9 code had a PPV of 63%. Using 85 SSc cases and 70 subjects that were not SSc cases, we calculated that that there would be 97% power to detect a PPV of 90% for an algorithm incorporating clinical data in addition to the SSc ICD-9 code with an alpha of 0.05 using Fisher’s exact test. Two-sided *p* values < 0.05 were considered to indicate statistical significance. Analyses were conducted using R version 3.5.1. Random numbers to select subjects for the training and validation sets were generated using R version 3.5.1 with a set seed of 1. The PS program (version 3.1.2) was used to compute sample size [9].

## Results

### Training set

An outline of our approach is shown in Fig. 1. Within the Synthetic Derivative, we identified 1899 possible SSc cases with at least 1 SSc ICD-9 or ICD-10-CM code. Of the 200 randomly selected subjects in the training set, 85 subjects were classified as true cases on chart review, 70 were defined as not SSc cases with alternative diagnoses, 24 had uncertainty in the SSc diagnosis, and 21 had missing specialist notes.

Of the 85 SSc cases, 4 were classified as having MCTD or overlap syndrome with SSc features. All subjects classified as cases were seen by either VUMC rheumatologists (*n* = 69) or VUMC pulmonologists (*n* = 16). Of the 70 subjects who were not cases, the most common diagnoses were systemic lupus erythematosus (SLE) (*n* = 38), Sjogren’s syndrome (*n* = 5), graft-versus-host disease (*n* = 5), morphea (*n* = 5), dermatomyositis (*n* = 4), rheumatoid arthritis (*n* = 4), undifferentiated connective tissue disease (*n* = 2), and *n* = 1 for each of the following: scleredema, scleromyxedema, positive scl-70 antibody, eosinophilic fasciitis, ankylosing spondylitis, tuberous sclerosis, and T cell lymphoma. Of the 24 subjects with uncertainty in the diagnosis, 6 were seen by a rheumatologist, 2 were seen by a dermatologist, and 2 were seen by a pulmonologist. The remainder of uncertain cases had mention of “scleroderma” or “CREST” in other specialists’ note(s) or listed in the past medical history of clinical notes. The 24 subjects with uncertain diagnoses were counted as “not cases” for analyses.

To ensure interrater reliability, a second rheumatologist (AB) chart reviewed 50 randomly selected subjects of the 200 subjects from the training set. Of these 50 randomly selected subjects, there was 92% agreement on case status between the 2 rheumatologists. The 4 subjects that the rheumatologists disagreed on had MCTD with SSc featulres and were counted as cases.

The 85 SSc cases and the 94 subjects (70 subjects without a SSc diagnosis and 24 subjects with uncertainty in diagnosis) who were counted as “not cases” are compared in Table 1. Compared to “not cases,” cases were older at time of data analysis (68 vs. 59, *p* <  0.01) with no differences in sex and race/ethnicity. Cases vs. “not cases” had higher counts of the SSc ICD-9 code (10 vs. 2, *p* <  0.01) and SSc ICD-10-CM codes (6 vs. 2, *p* <  0.01). “Not cases” had a longer duration of EHR follow-up (10 vs. 7 years, *p* <  0.01) compared to cases.

**Table 1:** Characteristics of SSc cases and non-cases in the training set

Characteristics	SSc cases (*n* = 86)	Non-cases (*n* = 94)	*p* value^1^
Age, years, mean ± standard deviation	68 ± 14	59 ± 20	< 0.01
Female, *n* (%)	71 (83%)	84 (89%)	0.19
White, *n* (%)	65 (76%)	70 (75%)	0.86
Number of counts of the SSc ICD-9^2^ code (710.1), mean ± standard deviation	10 ± 16	2 ± 5	< 0.01
Number of counts of the SSc ICD-10-CM^3^ codes (M34*), mean ± standard deviation	6 ± 7	2 ± 8	< 0.01
Years of follow-up^4^, mean ± standard deviation	7 ± 6	10 ± 7	< 0.01

^1^Mann-Whitney *U* test for continuous variables and chi-square test for categorical variables

^2^*ICD-9* International Classification of Diseases, Ninth Revision

^3^*ICD-10-CM* International Classification of Diseases, Tenth Revision, Clinical Modification

^4^Years of data available in the electronic health record from first to last ICD-9 and/or ICD-10-CM codes for any conditions

### ICD-9 algorithms

The performance of algorithms incorporating ICD-9 and ICD-10-CM codes with clinical data is shown in Table 2. As the number of counts of the SSc ICD-9 code increased, PPVs increased and sensitivities decreased. The PPV of ≥ 1 count of the ICD-9 code was 52%, 63% for ≥ 2 counts, 79% for ≥ 3 counts, and 86% for ≥ 4 counts. Incorporating a RP keyword with the ICD-9 code increased the PPVs. Adding ANA positivity to the ICD-9 code increased the PPVs.

**Table 2:** Performance of electronic health record algorithms for systemic sclerosis

Algorithm^1^	PPV (%)	Sensitivity (%)	*F*-score (%)
ICD-9 codes only			
≥ 1 count of the ICD-9 code (710.1)	52	100	81
≥ 2 counts	63	88	74
≥ 3 counts	79	72	75
≥ 4 counts	86	67	75
ICD-10 codes only			
≥ 1 count of the ICD-10 codes (M34*)	82	94	88
≥ 2 counts	84	91	87
≥ 3 counts	88	85	87
≥ 4 counts	91	85	88
ICD-9 or ICD-10 codes			
≥ 1 count	52	98	68
≥ 2 counts	70	97	81
≥ 3 counts	86	94	90
≥ 4 counts	91	91	91
ICD-9 code AND ANA positive^2^			
≥ 1 count of the ICD-9 codes AND ANA	53	81	64
≥ 2 counts of the ICD-9 codes AND ANA	68	81	74
≥ 3 counts of the ICD-9 codes AND ANA	84	70	77
≥ 4 counts of the ICD-9 codes AND ANA	93	64	76
ICD-10 codes AND ANA positive			
≥ 1 count of the ICD-10 codes AND ANA	95	53	68
≥ 2 counts AND ANA	95	53	68
≥ 3 counts AND ANA	100	50	67
≥ 4 counts AND ANA	100	50	67
ICD-9 code AND Raynaud’s (RP) keyword			
≥ 1 count of the ICD-9 code AND RP	78	90	84
≥ 2 counts AND RP	86	80	83
≥ 3 counts AND RP	92	66	77
≥ 4 counts AND RP	91	60	73
ICD-9 code, RP, ANA positive			
≥ 1 count of the ICD-9 code AND ANA OR RP	55	95	70
≥ 2 counts AND ANA OR RP	67	89	76
≥ 3 counts AND ANA OR RP	85	77	81
≥ 4 counts AND ANA OR RP	94	70	81
≥ 1 count AND ANA AND RP	75	75	75
≥ 2 counts AND ANA AND RP	87	75	80
≥ 3 counts AND ANA AND RP	94	66	77
≥ 4 counts AND ANA AND RP	93	59	72

^1^All algorithms included at least one or more counts of the SSc ICD-9 (710.1) or ICD-10-CM (M34*) codes for SSc

^2^ANA positive (titer ≥ 1:80)

We conducted a sensitivity analysis to determine the impact of date of the SSc ICD-9 code on the algorithms’ performances. We divided subjects in the training set using the date of their first SSc ICD-9 code in 5 year increments (1995–2000, 2000–2005, 2005–2010, 2010–2015). We then calculated the PPVs and sensitivities at each time period above for algorithms using ≥ 1 count, ≥ 2 counts, ≥ 3 counts, and ≥ 4 counts of the SSc ICD-9 code. We observed similar PPVs and sensitivities across the different time periods (Additional file 1: Table S1).

### ICD-10-CM algorithms

As the number of counts of the SSc ICD-10-CM codes increased, PPVs increased and sensitivities decreased (Table 2). The PPV of ≥ 1 count of the ICD-10-CM codes was 82%, 84% for ≥ 2 counts, 88% for ≥ 3 counts, and 91% for ≥ 4 counts. Algorithms using only the ICD-10-CM codes had higher PPVs compared to algorithms using only ICD-9 codes. Incorporating clinical data with the ICD-10-CM codes further improved the performance of the algorithms. Adding ANA positivity increased the PPVs. We did not have sufficient data because of the relatively low number of subjects with ICD-10-CM codes to calculate the performance of algorithms incorporating ICD-10-CM codes with RP keyword.

### ICD-9 or ICD-10-CM algorithms

As the number of counts of the SSc ICD-9 or ICD-10-CM codes increased, the PPVs increased with the sensitivities slightly decreasing (Table 2). The PPV of ≥ 1 count of either the ICD-9 or the ICD-10-CM codes was 52%, 70% for ≥ 2 counts, 86% for ≥ 3 counts, and 91% for ≥ 4 counts. PPVs for the algorithms using ICD-9 or ICD-10-CM codes were higher than the PPVs for algorithms using only ICD-9 codes but slightly lower than the algorithms using only ICD-10-CM codes. Sensitivities for the ICD-9 or ICD-10-CM algorithms did not decrease dramatically with higher counts of the codes compared to algorithms using only ICD-9 codes.

### Classification and regression tree and random forest modeling

In addition, we employed classification and regression tree (CART) and random forest (RF) modeling techniques for algorithm development. For CART modeling, the algorithm with the highest PPV included the following as nodes: RP keyword, SSc ICD-9 code counts, and SSc ICD-10-CM code counts with a PPV of 82% and a sensitivity of 85%. For RF, the algorithm with the highest PPV included 500 trees and sampled 2 random variables per tree with a PPV of 84%, a sensitivity of 92%, and an *F*-score of 88%. The most important feature for this algorithm was the presence of a RP keyword.

### High-performing algorithms

Algorithms using ≥ 3 or ≥ 4 counts of the SSc ICD-9 or ICD-10-CM codes and ANA positivity had the highest PPV of 100%. These algorithms, however, had a low sensitivity of 50%. The algorithm with the highest *F*-score of 91% was ≥ 4 counts of the ICD-9 or ICD-10-CM codes. This algorithm had a PPV of 90% in the validation set. When we applied this algorithm to the entire Synthetic Derivative with over 3 million subjects, we found 731 possible SSc subjects.

### Excluding SLE

As most of the non-case subjects had a diagnosis of systemic lupus erythematosus (SLE), we examined the effects of excluding subjects with at least one count of the SLE ICD-9 or ICD-10-CM codes on the algorithms’ PPVs and sensitivities. All 38 of the non-case subjects with a diagnosis of SLE had codes for both SLE and SSc. Overall, the PPVs of algorithms did not significantly increase when the SLE ICD-9 or ICD-10-CM code was excluded but sensitivities decreased (Additional file 1: Table S2).

### Using SSc classification criteria

We conducted a sensitivity analysis using the 2013 American College of Rheumatology/European League Against Rheumatism (ACR/EULAR) criteria [10] as the case definition for SSc rather than specialist diagnosis. Of the 85 SSc cases, 59 subjects (69%) fulfilled classification criteria and 27 subjects (32%) had missing clinical documentation to determine if they fulfilled classification criteria. Only one subject in the training set considered not a case during initial analysis was defined as a SSc case based on classification criteria. This subject was followed by gastroenterology and not one of the specialists required as part of our case definition but had documentation throughout the clinical notes to fulfill the 2013 ACR/EULAR criteria [10].

### Chart review of SSc cases

We chart reviewed the 85 true SSc cases from the training set and the 53 true SSc cases from the validation set. Of the 138 subjects, 127 had available documentation to determine SSc subset, of which 80% (*n* = 102) were limited and 20% (*n* = 25) diffuse. Of the 127 subjects with available documentation, 30% (*n* = 38) had pulmonary arterial hypertension diagnosed by a specialist (i.e., rheumatology or pulmonary) and 31% (*n* = 39) had interstitial lung disease diagnosed by a specialist. Mean estimated SSc duration was 4.5 years, defined as time from first SSc billing code to last clinical encounter in the EHR of any kind.

## Discussion

We developed and validated EHR-based algorithms with high PPVs that combined billing codes with clinical data to identify SSc patients. To the best of our knowledge, we are the first to develop and validate algorithms that combine billing codes with clinical data to identify SSc patients within the EHR. These algorithms are important, as they identify SSc patients not just in the rheumatology clinic but across the healthcare system. With a rare disease such as SSc, identifying all potential patients is critical to enable studies to have power to examine outcomes.

One study only examined the performance of using 1 count of the SSc ICD-9 code to identify SSc cases in the EHR and reported a PPV of 76% [3]. This PPV may be higher compared to our 52%, as SSc cases were defined differently. In contrast to our case definition requiring a specialist’s diagnosis, Valenzuela et al. defined SSc cases as patients who fulfilled one of the three SSc classification criteria [3]. Further, Valenzuela et al. did not validate their findings or investigate higher counts of the SSc billing codes [3]. Redd et al. also examined methods to identify SSc in the EHR, specifically within the Veterans Health Administration database [2]. The objective of their study differed as they aimed to identify patients at risk for scleroderma renal crisis. They applied natural language processing (NLP) using keywords such as “systemic sclerosis” or “scleroderma” and keywords that captured SSc clinical features such as “Raynaud’s phenomenon” and “digital tip ulcers” to assemble their SSc cohort. ANA positivity was not included in their retrieval criteria. While NLP can be an efficient way to capture data, it often requires programming and bioinformatics support that may not be available at all institutions. Redd et al. also did not perform a true validation of the retrieval criteria used in their NLP-based algorithm [2]. Lastly, a recent retrospective cohort study from Gordon et al. looked at risk factors for SRC at time of SSc diagnosis [11]. They also assembled their cohort using 1 count of the SSc ICD-9 code and then chart reviewed all the subjects (*n* = 749) to determine case status with a PPV of 62%. This PPV was slightly higher than our PPV of 53% likely due to using a different SSc case definition, which was either a documented diagnosis of SSc by a rheumatologist or meeting the ACR/EULAR 2013 classification criteria [10]. Using an algorithm or search strategy that requires comprehensive chart review can be time consuming and potentially not feasible for some studies. Gordon et al. also used a military database which may limit the generalizability of their findings [11]. Similar to the other two studies above, a validation was not performed on the performance of the algorithm.

While the above studies used the SSc ICD-9 code to assemble their SSc cohorts, we sought to develop algorithms that also incorporated ICD-10-CM codes, particularly with the widespread implementation of ICD-10-CM codes in the USA as of October 2015. The PPVs were higher for algorithms only using ICD-10-CM codes compared to algorithms only using ICD-9 codes. One possible explanation is that ICD-10-CM codes are more comprehensive compared to ICD-9 codes in that they require specification of organ involvement and type of SSc. This increased level of specificity in the coding may discourage clinicians from using in subjects who may not fit clinical criteria for SSc or where there is uncertainty in the diagnosis. Another hypothesis for the higher PPVs with the ICD-10-CM code algorithms is that early adopters of the ICD-10-CM codes are specialists who routinely encounter patients with SSc, particularly rheumatologists. It would be expected that these specialists would then use the ICD-10-CM codes more appropriately in labeling SSc patients compared to clinicians who rarely see or manage SSc. Thus, the ICD-10-CM codes may not necessarily be superior to ICD-9 codes but are just used more frequently by specialists, such as rheumatologists.

In addition to developing algorithms using ICD-10-CM codes, we also added clinical data to algorithms. As our results suggest, relying solely on 1 or 2 counts of the SSc ICD-9 code may not be sufficient to identify SSc subjects accurately in the EHR. Adding ANA positivity and a RP keyword both significantly improved the PPVs of the algorithms compared to algorithms using only SSc ICD-9 or ICD-10-CM codes. A priori, we discussed with a panel of rheumatologists clinical features that could be captured as keywords in the EHR to add as components to the algorithms. We chose a RP keyword, as over 95% of patients with SSc have RP [12]. Other signs or symptoms discussed for potential use in the algorithms were gastroesophageal reflux, dysphagia, interstitial lung disease (ILD), and pulmonary arterial hypertension (PAH). As there is disease heterogeneity in SSc, with some patients having ILD or PAH and others not, requiring these clinical features might exclude subjects that truly have SSc. Further, conditions such as reflux or dysphagia are not specific to SSc and use of these clinical features in an algorithm could potentially capture subjects without SSc.

As expected, the more office visits a potential SSc patient has, the more times a billing code for SSc is used with the clinician feeling confident with that billing code’s diagnosis. Thus, the higher the counts or more uses of the ICD-9 and ICD-10-CM codes, the higher the PPVs. Using algorithms that require higher counts of billing codes could potentially exclude critically ill patients with SSc (i.e., renal crisis) who die during their initial presentation. Requiring higher counts of billing codes could also capture SSc patients with more severe disease and organ involvement. These patients would be seen more frequently and would likely require multiple specialists to co-manage their disease. As our database is de-identified, we do not have access to insurance status to assess its impact on algorithms’ performances.

While algorithms with high PPVs would more accurately capture true cases, this is usually at the expense of a lower sensitivity. Our goal was to capture SSc not only in the rheumatology clinic, but also across the healthcare system, including SSc patients that follow with other specialists. Thus, we chose to use and validate the algorithm with the highest *F*-score, which accounts for both PPV and sensitivity. A high *F*-score maximizes both the PPV and sensitivity to increase accuracy that a subject is a true SSc case while casting a wide enough net to capture as many SSc subjects in the EHR as possible. The algorithm with the highest *F*-score also incorporates both ICD-9 and ICD-10-CM codes, which would capture both historical and current subjects in the EHR.

We also used machine learning methods to generate algorithms. While these algorithms were not the highest performing in terms of PPV, they represent an alternative approach to algorithm development. Rule-based algorithms (i.e., algorithms connecting data elements using “and” or “or”) are straightforward to develop and do not require expertise in computational models. Since they are based on user input variables, they do require pre-existing knowledge of important classifier variables. These algorithms can be rather time-intensive to develop and validate, and they require trial and error to find the model with the lowest error. In contrast, machine learning methods often do not require specific domain knowledge. A major strength of these methods is that models can be tuned automatically to easily identify the optimal model parameters that maximize sensitivity and PPV. Algorithms generated by machine learning methods such as random forests allow for a robust, data-driven approach that can model more complex interactions without sacrificing power in contrast to rule-based algorithms that use simple Boolean logic such as “and” or “or” to combine data elements.

Investigators may select algorithms based on study goals and available data. If the goal of the study is to ensure subjects have SSc with high certainty, for example a genetics study, then one would select an algorithm with a high PPV to increase the chances that the subjects are true SSc cases. In contrast, if one wants to capture as many SSc subjects as possible to ensure an adequate sample size to study a rare outcome, one would select an algorithm with a high sensitivity. Additional concerns in selecting an algorithm would be if chart review is readily available and feasible. If so, one might select an algorithm with a higher sensitivity but not the highest PPV. This approach would allow a broad capture of all possible SSc subjects with the ability to chart review ensuring subjects are true SSc cases. If chart review is not desired or feasible, one would select an algorithm with a high PPV to ensure a high proportion of subjects are true SSc cases. Further, if an institution does not have access to clinical data, they could select an algorithm that uses only billing codes.

While we developed and validated high-performing algorithms that combined billing codes with clinical data to identify SSc subjects in the EHR, there are limitations to our study. To start our search for SSc subjects within Vanderbilt’s Synthetic Derivative of over 3 million subjects, we used a one-time count of either the SSc ICD-9 or SSc ICD-10-CM codes. It is possible that a SSc subject could have been missed that did not have at least one SSc billing code. To test this hypothesis, we searched for potential SSc subjects without a SSc ICD-9 or ICD-10-CM codes but who had a keyword of “systemic sclerosis,” or “scleroderma,” in the clinical record to approximate a negative predictive value (NPV) for algorithms using SSc ICD-9 or ICD-10-CM codes. We then randomly selected 100 of these potential SSc subjects for chart review to determine case status. We found only 1 subject who had SSc on chart review estimating the NPV to be 99%. This subject had juvenile dermatomyositis (DM) with scleroderma features but had only DM billing codes.

We developed and internally validated our algorithms at a single academic institution potentially limiting portability and generalizability of our algorithms. Similar studies for EHR-based algorithms in rheumatoid arthritis, however, have demonstrated good portability [13]. Further, our internal validation was successful with a PPV of 90% in our validation set compared to 91% in our training set for the algorithm with the highest *F*-score. Future steps would be to externally validate our algorithms within other institutions’ EHRs. Lastly, we used a case definition for SSc based on a specialist’s diagnosis and not SSc clinical criteria. We found missingness in the systematic documentation of SSc criteria in the clinical notes, as we have also reported in SLE [4]. Using the SSc criteria as the case definition would then result in true SSc subjects being labeled as not cases, particularly subjects without detailed specialists’ notes. Further, missingness in clinical documentation on type of SSc limited our ability to determine how algorithms performed in identifying limited vs. diffuse cutaneous SSc patients.

While the EHR cannot substitute for a prospective cohort study, it can increase the efficiency of a study and dramatically increase sample size. For example, at our institution, it took 2 years to enroll approximately 60 SSc subjects into a prospective cohort. In contrast, deploying 1 of our high-performing algorithms of ≥ 4 counts of the SSc ICD-9 or ICD-10-CM codes with a PPV of 90% resulted in 731 possible SSc subjects.

## Conclusions

Accurately identifying SSc patients in the EHR enables researchers to assemble a large and diverse cohort of SSc patients with decreased cost and increased efficiency compared to traditional cohort studies. While administrative databases are cost efficient and allow for high powered studies, their validity is limited by the accuracy of billing codes. Administrative databases may also lack detailed clinical data such as labs, radiology, and pathology. By bridging the gaps between administrative databases and prospective cohort studies, EHR-based cohorts represent powerful tools. Researchers can accurately and efficiently identify SSc subjects, examine outcomes longitudinally, and ask clinically important questions about the disease.

## Supplementary information

**Additional file 1: Table S1.** Performance of ICD-9 billing code algorithms by date. **Table S2.** Performance of electronic health record algorithms for systemic sclerosis excluding codes for systemic lupus erythematosus.

## Data Availability

The data used and analyzed during the current study are available from the corresponding author on reasonable request. Note that row level data is access controlled and cannot be provided publicly due to data use restrictions.

## Abbreviations

ACR: American College of Rheumatology
ANA: Antinuclear antibody
CART: Classification and regression tree
EHR: Electronic health record
EULAR: European League against Rheumatism
ICD: International Classification of Diseases
ILD: Interstitial lung disease
MCTD: Mixed connective tissue disease
NLP: Natural language processing
PAH: Pulmonary arterial hypertension
PPV: Positive predictive value
RF: Random forests
RP: Raynaud’s phenomenon
SLE: Systemic lupus erythematosus
SSc: Systemic sclerosis
VUMC: Vanderbilt University Medical Center
