# Supplementary material for: Rule-based and machine learning algorithms identify patients with systemic sclerosis accurately in the electronic health record
Source: Arthritis Res Ther. 2019 Dec 30;21:305. doi: 10.1186/s13075-019-2092-7 (PMC6937803; doi:10.1186/s13075-019-2092-7)
Supplement: Supplementary file 1 — Additional file 1: Table S1. Performance of ICD-9 billing code algorithms by date. Table S2. Performance of electronic health record algorithms for systemic sclerosis excluding codes for systemic lupus erythematosus. [file 13075_2019_2092_MOESM1_ESM.docx]

**Table S1. Performance of ICD-9 billing code algorithms by date.**

| **Years^1^** | **ICD-9 code counts** | **Positive Predictive Value** | **Sensitivity** |
| --- | --- | --- | --- |
| 1995 - 2000  (n = 9) | ≥ 1  ≥ 2  ≥ 3  ≥ 4 | 0.50  0.57  0.75  0.67 | 1.00  1.00  0.75  0.50 |
| 2000 – 2005  (n = 47) | ≥ 1  ≥ 2  ≥ 3  ≥ 4 | 0.66  0.77  0.88  0.93 | 1.00  0.97  0.90  0.87 |
| 2005 – 2010  (n = 70) | ≥ 1  ≥ 2  ≥ 3  ≥ 4 | 0.53  0.64  0.79  0.88 | 1.00  0.89  0.60  0.60 |
| 2010 – 2015  (n = 59) | ≥ 1  ≥ 2  ≥ 3  ≥ 4 | 0.51  0.60  0.83  0.85 | 0.97^2^  0.86  0.66  0.59 |

^1^Date of first SSc ICD-9 code (710.1).

^2^There was a transition period when both ICD-9 and ICD-10-CM codes were in use. The sensitivity is not 100% as some patients were coded with only ICD-10-CM codes prior to the full transition from ICD-9 to ICD-10-CM.

**Table S2. Performance of electronic health record algorithms for systemic sclerosis excluding codes for systemic lupus erythematosus.**

| **Algorithm**^1^ | **PPV** | **PPV excluding**  **SLE^2^** | **Sensitivity** | **Sensitivity excluding**  **SLE** | **F-score** | **F-score excluding SLE** |
| --- | --- | --- | --- | --- | --- | --- |
| **ICD-9 codes only** |  |  |  |  |  |  |
| ≥ 1 count of the ICD-9 code (710.1) | 52% | 56% | 98% | 72% | 68% | 63% |
| ≥ 2 counts | 63% | 63% | 88% | 53% | 74% | 58% |
| ≥ 3 counts | 79% | 75% | 72% | 47% | 75% | 58% |
| ≥ 4 counts | 86% | 80% | 67% | 38% | 75% | 52% |
| **ICD-10 codes only** |  |  |  |  |  |  |
| ≥ 1 count of the ICD-10 codes | 82% | 73% | 94% | 51% | 88% | 60% |
| ≥ 2 counts | 84% | 80% | 91% | 43% | 87% | 56% |
| ≥ 3 counts | 88% | 85% | 85% | 30% | 87% | 44% |
| ≥ 4 counts | 91% | 90% | 85% | 24% | 88% | 38% |
| **ICD-9 or ICD-10 codes** |  |  |  |  |  |  |
| ≥ 1 count | 52% | 56% | 98% | 72% | 68% | 63% |
| ≥ 2 counts | 70% | 58% | 97% | 57% | 81% | 58% |
| ≥ 3 counts | 86% | 73% | 94% | 43% | 90% | 54% |
| ≥ 4 counts | 91% | 87% | 91% | 35% | 91% | 50% |

^1^All algorithms included at least one or more counts of the SSc ICD-9 (710.1) or ICD-10-CM (M34*) codes.

^2^Excluding subjects with SLE ICD-9 (710.0) or SLE ICD-10-CM (M32.1, M32.8, and M32.9) codes.
